# An international multicenter retrospective study of *Pseudomonas aeruginosa* nosocomial pneumonia: impact of multidrug resistance

**DOI:** 10.1186/s13054-015-0926-5

**Published:** 2015-05-06

**Authors:** Scott T Micek, Richard G Wunderink, Marin H Kollef, Catherine Chen, Jordi Rello, Jean Chastre, Massimo Antonelli, Tobias Welte, Bernard Clair, Helmut Ostermann, Esther Calbo, Antoni Torres, Francesco Menichetti, Garrett E Schramm, Vandana Menon

**Affiliations:** St. Louis College of Pharmacy, 4588 Parkview Place, St. Louis, MO 63110-1088 USA; Northwestern University Feinberg School of Medicine, McGaw Pavilion Suite M-300, 240 E Huron, Chicago, IL 60611 USA; Division of Pulmonary and Critical Care Medicine, Washington University School of Medicine, 660 South Euclid Avenue, Campus Box 8052, St. Louis, MO 63110 USA; Vall d’Hebron University Hospital, Passeig Vall d’Hebron, 119, Barcelona, 08035 Spain; Service de Réanimation Médicale, Institut de Cardiologie, Groupe Hospitalier Pitié-Salpêtrière, 47-83 boulevard de l’Hôpital, 75651 Paris, Cedex 13 France; Policlinico Universitario A Gemelli, Largo Agostino Gemelli 8, Rome, 00168 Italy; Medizinische Hochschule, Carl-Neuberg-Str. 1, Hannover, 30625 Germany; Hôpital Raymond Poincaré, 104 boulevard Raymond Poincaré, Garches, 92380 France; Department of Hematology, University Hospital Grosshadern, Marchioninistr 15, Munich, D-81377 Germany; Hospital Universitari MútuaTerrassa, Plaça Dr. Robert, 5, Terrassa, 08221 Spain; Pneumology Department, Clinic Institute of Thorax, Villarroel 170, Barcelona, 08036 Spain; Malattie Infettive, Az. Ospedaliera Universitaria Pisana, Via Paradisa 2 – Cisanello, Pisa, 56100 Italy; Pharmacy Services, Mayo Clinic, 200 First St SW, Rochester, MN 55905 USA; Cubist Pharmaceuticals, Inc., 65 Hayden Avenue, Lexington, MA 02421 USA

## Abstract

**Introduction:**

*Pseudomonas aeruginosa* nosocomial pneumonia (*Pa*-NP) is associated with considerable morbidity, prolonged hospitalization, increased costs, and mortality.

**Methods:**

We conducted a retrospective cohort study of adult patients with *Pa*-NP to determine 1) risk factors for multidrug-resistant (MDR) strains and 2) whether MDR increases the risk for hospital death. Twelve hospitals in 5 countries (United States, n = 3; France, n = 2; Germany, n = 2; Italy, n = 2; and Spain, n = 3) participated. We compared characteristics of patients who had MDR strains to those who did not and derived regression models to identify predictors of MDR and hospital mortality.

**Results:**

Of 740 patients with *Pa*-NP, 226 patients (30.5%) were infected with MDR strains. In multivariable analyses, independent predictors of multidrug-resistance included decreasing age (adjusted odds ratio [AOR] 0.91, 95% confidence interval [CI] 0.96-0.98), diabetes mellitus (AOR 1.90, 95% CI 1.21-3.00) and ICU admission (AOR 1.73, 95% CI 1.06-2.81). Multidrug-resistance, heart failure, increasing age, mechanical ventilation, and bacteremia were independently associated with in-hospital mortality in the Cox Proportional Hazards Model analysis.

**Conclusions:**

Among patients with *Pa*-NP the presence of infection with a MDR strain is associated with increased in-hospital mortality. Identification of patients at risk of MDR *Pa*-NP could facilitate appropriate empiric antibiotic decisions that in turn could lead to improved hospital survival.

## Introduction

Recent trends show an increase in the prevalence of nosocomial pneumonia (NP) caused by multidrug-resistant (MDR) Gram-negative bacteria, most commonly *Pseudomonas aeruginosa* with documented resistance to β-lactams, carbapenems, aminoglycosides, and fluoroquinolones [1-3]. Consequently, the therapeutic effectiveness of current therapies for bacterial NP is becoming increasingly limited, emphasizing the need for development of new and effective antimicrobials as well as novel strategies to prevent resistance emergence [4,5].

Nosocomial pneumonia due to *P. aeruginosa* (*Pa*-NP) is associated with considerable morbidity, prolonged hospitalization, increased costs, and mortality [6-8]. *P. aeruginosa* is one of the few pathogens independently associated with increased mortality among patients with sepsis or pneumonia in the ICU setting [6,9]. The mortality associated with *Pa*-NP is further increased when inappropriate initial antibiotic therapy (IIAT) is prescribed, usually due to the presence of MDR pathogens [10-13]. The overall impact of *Pa*-NP on clinical outcomes and healthcare costs underscores the importance of this nosocomial infection. Therefore, we performed a multinational study with the following objectives: first, to evaluate the prevalence of MDR *Pa*-NP and to identify clinical risk factors associated with MDR *Pa*-NP; second, to evaluate the influence of MDR status on patient outcomes.

## Methods

### Study design and ethical standards

We conducted a retrospective study in 12 hospitals in 5 countries (United States, n = 3; France, n = 2; Germany, n = 2; Italy, n = 2; and Spain, n = 3). Eligible patients were aged ≥18 years consecutively admitted for their index hospitalization within 36 months prior to study initiation in 2013. All eligible patients met a clinical diagnosis of NP defined as new or progressive infiltrates consistent with pneumonia on chest radiograph or computed tomography and either a temperature >38.3°C or leukocytosis >10,000 cells/mm^3^ or both. To be eligible, patients had to have *P. aeruginosa* cultured from at least one of the following respiratory specimens, including sputum, pleural fluid, flexible bronchoscopy with protected specimen brush, bronchoalveolar (BAL), transbronchial biopsy, nonbronchoscopic BAL, or tracheobronchial aspirate in intubated patients. Microbiologic cultures (qualitative or quantitative) had to be obtained within the 12-hour window before or the 12-hour window after the initiation of antibiotic(s) targeting *P. aeruginosa*. Each investigator obtained approval and a waiver of patient consent from an Independent Ethics Committee or Institutional Review Board at their institution before commencing the study. The list of all ethical bodies that approved the study can be found in the Acknowledgements section.

### Endpoints and covariates

The primary endpoints examined were multidrug-resistance and hospital mortality. We collected important covariates including demographics, comorbidities (heart failure, diabetes mellitus, chronic obstructive pulmonary disease, chronic kidney disease, chronic liver disease, hematologic malignancy, solid tumor, HIV/AIDS, and dementia). In addition, important process-of-care variables, including ICU admission, mechanical ventilation, vasopressor administration, and the appropriateness of initial antibiotic therapy, were collected.

### Definitions

To be classified as MDR, the *P. aeruginosa* isolate had to be non-susceptible to one or more agents in three or more of the following antimicrobial categories, as determined by the European Center for Disease Prevention and Control (ECDC) and the Centers for Disease Control and Prevention (CDC): aminoglycosides, antipseudomonal carbapenems, antipseudomonal cephalosporins, antipseudomonal fluoroquinolones, antipseudomonal penicillins plus β-lactamase inhibitors, monobactams, phosphonic acids, and polymixins. To be classified as extensively drug-resistant (XDR), the *P. aeruginosa* isolate had to be non-susceptible to one or more agents in all but two or more of the aforementioned antimicrobial categories [14]. Antimicrobial treatment was deemed to be appropriate (AIAT) if at least one of the initially prescribed antibiotics was active against the identified *P. aeruginosa* isolate based on *in vitro* susceptibility testing and this antibiotic was administered within 24 hours after collection of the respiratory specimen [15].

### Antimicrobial susceptibility testing

Microbiology laboratories performed antimicrobial susceptibility testing of isolates using disk diffusion or automated testing methods according to guidelines and breakpoints established by the Clinical Laboratory and Standards Institute (CLSI) [16] and the European Committee on Antimicrobial Susceptibility Testing (EUCAST) [17].

### Statistical analyses

Continuous variables were reported as means with standard deviation or the median and interquartile range from non-normally distributed data. Differences between continuous variables were tested using Student’s *t*-test or the nonparametric Mann-Whitney *U*-test. Categorical data were summarized as proportions, and the Chi-square test or Fisher’s exact test for small samples was used to examine differences between groups. Univariate and multivariate logistic regression models were constructed to identify clinical risk factors associated with multidrug-resistance. All variables that showed a significant result in the univariate analysis (≤0.10) were included in the corresponding multivariate logistic regression analysis. All variables entered into the models were examined to assess for co-linearity, and interaction terms were tested. The model’s calibration was assessed with the Hosmer-Lemeshow goodness-of-fit test. A Cox proportional hazards model was constructed to determine variables independently associated with hospital mortality. This test was selected to exclude the influence of time-dependent covariates on hospital mortality and to adequately control for imbalances in baseline and clinical characteristics when constructing a survival curve. All tests were two-tailed, and a *P*-value <0.05 was deemed a priori to represent statistical significance. All analyses were performed with SPSS software, version 19.0 (IBM SPSS, Chicago, IL, USA).

## Results

Seven hundred and forty patients with *Pa*-NP met the inclusion criteria and were enroled in the study: 258 (34.9%) from the United States, 141 (19.1%) from France, 120 (16.2%) from Germany, 113 (15.3%) from Spain and 108 (14.6%) from Italy. The prevalence of multidrug resistance was 30.5%. The patients’ baseline and clinical characteristics are shown in Table 1. Patients with pneumonia caused by MDR strains of *P. aeruginosa* were significantly younger and were more likely to be admitted to the hospital from an inpatient rehabilitation facility compared to patients infected with non-MDR strains. Patients with MDR strains were significantly more likely to have received antibiotics in the 30 days prior to the diagnosis of pneumonia and were also more likely to have chronic obstructive pulmonary disease and diabetes mellitus. A significantly higher proportion of patients who were infected with an MDR strain received IIAT (37.9% versus 19.2%, *P* <0.001) and required ICU admission (79.6% versus 71.4%, *P* = 0.019) compared to those with a non-MDR strain.

**Table 1:** **Clinical and epidemiological characteristics of multidrug (MDR) and non-multidrug resistant patients with** ***Pseudomonas aeruginosa*** **pneumonia**

**Characteristic**	**Percent missing (of total 740)**	**MDR N = 226**	**Non-MDR N = 514**	***P*** **-value**
Age, years, mean ± SD	0.5%^a^	53.5 ± 17.5	62.1 ± 15.5	<0.001
Male	0%	142 (62.8%)	361 (70.2%)	0.047
Location prior to admission	1.1%			
Community		101 (44.7%)	286 (55.6%)	0.006
Skilled nursing facility		17 (7.5%)	37 (7.2%)	0.876
Long-term care facility		7 (3.1%)	20 (3.9%)	0.596
Assisted living		4 (1.8%)	3 (0.6%)	0.125
Inpatient rehabilitation		27 (11.9%)	20 (3.9%)	<0.001
Other		66 (29.2%)	144 (28.0%)	0.741
Past medical history				
Hospitalized in the previous 6 months	13.1%	126 (60.6%)	245 (56.3%)	0.307
Antibiotics in the previous 30 days	27.6%	100 (57.5%)	163 (45.0%)	0.007
Heart failure	9.6%	49 (23.2%)	131 (28.6%)	0.145
Chronic obstructive pulmonary disease	9.2%	102 (48.8%)	173 (37.4%)	0.005
Diabetes mellitus	8.6%	79 (37.8%)	137 (29.3%)	0.029
Chronic kidney disease	9.3%	55 (26.3%)	118 (25.5%)	0.832
Chronic liver disease	11.9%	38 (18.5%)	70 (15.7%)	0.359
Hematologic malignancy	9.9%	20 (9.4%)	40 (8.8%)	0.807
Solid tumor	10.3%	18 (8.7%)	81 (17.7%)	0.002
HIV/AIDS	10.5%	3 (1.5%)	6 (1.3%)	0.885
Dementia	12.6%	6 (3.0%)	36 (8.1%)	0.015
Charlson score, mean ± SD	2.6%	3.1 ± 2.6	3.0 ± 2.6	0.869
Pneumonia category	0%			
Community-onset, healthcare-associated		74 (32.7%)	167 (32.5%)	0.946
Hospital-onset		152 (67.2%)	347 (67.5%)	0.946
Hospital-acquired		50 (22.1%)	112 (21.8%)	0.919
Ventilator-associated		102 (45.1%)	235 (45.7%)	0.883
ICU admission	0%	180 (79.6%)	367 (71.4%)	0.019
Length of ICU stay, days, median (IQR)	0%	18.9 (11.4, 32.5)	16.1 (8.7, 29.1)	0.058
Mechanical ventilation	0%	197 (87.2%)	440 (85.6%)	0.571
Length of mechanical ventilation, days, median (IQR)	0%	17.0 (9.1, 34.1)	13.1 (6.5, 26.0)	0.006
Vasopressor administration	0%	146 (64.6%)	308 (59.9%)	0.229
Bacteremia	0%	53 (23.5%)	128 (24.9%)	0.672
Inappropriate initial antibiotic therapy	1.5%	83 (37.9%)	98 (19.2%)	<0.001
In-hospital mortality	0%	101 (44.7%)	163 (31.7%)	0.001
Length of hospital stay, days, median (IQR)	0%	27.0 (14.0, 56.3)	25.0 (13.0, 46.0)	0.090

^a^Four patients aged >90 years (one MDR, three non-MDR) were not included in the calculation.

Susceptibility to all antibiotic classes tested was significantly lower in patients infected with MDR strains (Table 2). Antibiotic susceptibility by country is found in Table 3. Germany (44.2%) and Spain (43.4%) were found to have the highest prevalence of MDR, followed by France (33.3%), Italy (22.2%) and the United States (20.5%). Table 4 shows the results of a multivariable logistic regression model that identified the variables associated with pneumonia caused by MDR strains of *P. aeruginosa*. Decreasing age in increments of one year, diabetes mellitus, and ICU admission were independently associated with MDR *P. aeruginosa* pneumonia.

**Table 2:** **Antibiotic susceptibility**

**Antibiotic class**	**Multidrug-resistant (n = 226)**	**Non-multidrug-resistant (n = 514)**	***P*** **-value**
Aminoglycosides	226 (29.2%)	505 (91.1%)	<0.001
Antipseudomonal carbapenems	226 (15.0%)	508 (84.6%)	<0.001
Antipseudomonal cephalosporins	226 (26.5%)	504 (93.7%)	<0.001
Antipseudomonal fluoroquinolones	222 (21.5%)	502 (88.4%)	<0.001
Antipseudomonal penicillins +	221 (22.2%)	502 (89.0%)	<0.001
β-lactamase inhibitors			
Monobactams	158 (13.9%)	208 (81.2%)	<0.001
Phosphonic acids	86 (40.7%)	105 (81.0%)	<0.001
Polymyxins	159 (97.5%)	215 (92.1%)	0.025

Data presented as number of isolates tested (% susceptible). Multidrug-resistant: non-susceptible to one or more agents in three or more antibiotic classes.

**Table 3:** **Antibiotic susceptibility by country**

**Antibiotic class**	**France**	**Germany**	**Italy**	**Spain**	**United States**
Aminoglycosides	141(76.6)	120 (58.3)	101 (75.2)	112 (58.9)	257 (80.2)
Antipseudomonal carbapenems	139 (60.4)	119 (52.1)	107 (57.9)	112 (47.3)	257 (79.0)
Antipseudomonal cephalosporins	140 (77.1)	120 (60.8)	101 (74.3)	111 (59.5)	258 (81.4)
Antipseudomonal fluoroquinolones	138 (66.7)	118 (61.0)	100 (75.0)	111 (52.3)	257 (75.9)
Antipseudomonal penicillins +	141 (64.5)	118 (46.5)	108 (70.3)	110 (63.6)	253 (82.6)
β-lactamase inhibitors					
Multidrug-resistant	141 (33.3)	120 (44.2)	108 (22.2)	113 (43.4)	258 (20.5)
Extensively drug-resistant	141 (17.7)	120 (34.2)	108 (2.8)	113 (13.3)	258 (3.5)

Data presented as number of isolates tested (% susceptible). Multidrug-resistant: non-susceptible to one or more agents in three or more antibiotic classes. Extensively drug-resistant: non-susceptible to one or more agents in all but two or fewer antibiotic classes.

**Table 4:** **Significant univariate and multivariate logistic regression analysis of predictors for multidrug-resistant (MDR)** ***Pseudomonas aeruginosa*** **pneumonia**

	**Univariate**		**Multivariate** ^**a**^	
**Variable**	**Odds ratio (95% CI)**	***P*** **-value**	**Odds ratio (95% CI)**	***P*** **-value**
Age (decreasing increments of 1)	0.97 (0.96, 0.98)	<0.001	0.97 (0.96, 0.98)	<0.001
Male	0.72 (0.52, 0.99)	0.047		
Residence in a community setting prior to admission	0.64 (0.47, 0.88)	0.006		
Residence in an inpatient rehabilitation facility prior to admission	3.35 (1.84, 6.11)	<0.001		
Antibiotics in the previous 30 days	1.65 (1.15, 2.38)	0.007		
Chronic obstructive pulmonary disease	1.60 (1.15, 2.22)	0.005		
Diabetes mellitus	1.46 (1.04, 2.06)	0.030	1.90 (1.21, 3.00)	0.006
Solid tumor	0.44 (0.26, 0.76)	0.003		
Dementia	0.35 (0.15, 0.85)	0.020		
ICU admission	1.57 (1.08, 2.28)	0.019	1.73 (1.06, 2.81)	0.028

^a^Hosmer-Lemeshow goodness-of-fit test, *P* = 0.72.

**Figure 1:**
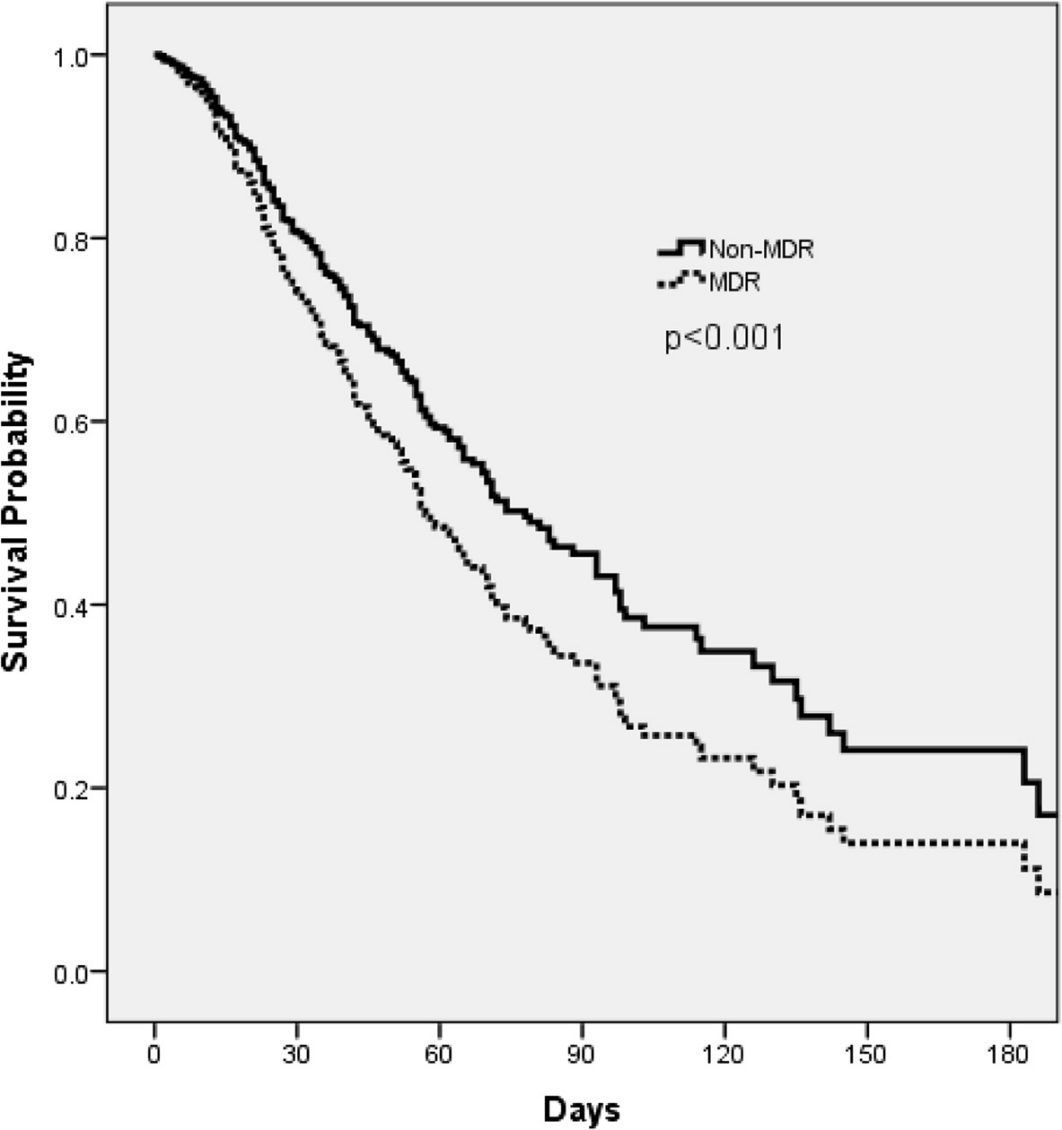
Cox proportional hazards model curve comparing patients with multidrug-resistant (MDR)-*Pseudomonas aeruginosa* and those with non-MDR *P. aeruginosa* nosocomial pneumonia.

The overall, hospital mortality was 35.7% (n = 264). Mortality was significantly different between the United States and European countries: United States, 22.5%; France, 37.6%; Germany, 41.7%; Spain, 46.9%; and Italy, 46.3%. Patients with MDR strains had a significantly higher in-hospital mortality rate compared to non-MDR infected patients (Table 1). A Cox proportional hazards model confirmed MDR status as an independent predictor of mortality (hazard ratio (HR) 1.39, 95% CI 1.05 to 1.83, *P* = 0.021) along with increasing age, heart failure, concomitant bacteremia, mechanical ventilation, and patients residing in Germany, Italy, and Spain (Table 5). Cox model-adjusted survival curve analysis controlling baseline and clinical imbalances confirmed the influence of MDR on in-hospital mortality (Figure 1).

**Table 5:** **Cox proportional hazards model of significant predictors for in-hospital mortality**

**Variable**	**Hazards ratio (95% CI)**	***P*** **-value**
Heart failure	1.88 (1.39, 2.52)	<0.001
Age (increasing increments of 1 year)	1.02 (1.01, 1.03)	0.001
Country of origin, Germany	3.05 (1.87, 4.96)	<0.001
Country of origin, Italy	2.38 (1.41, 4.02)	0.001
Country of origin, Spain	1.91 (1.16, 3.14)	0.011
Mechanical ventilation	1.88 (1.02, 3.48)	0.044
Bacteremia	1.67 (1.20, 2.31)	0.002
Multidrug resistance	1.39 (1.05, 1.83)	0.021
No vasopressors	0.61 (0.43, 0.87)	0.006
Healthcare associated pneumonia	0.50 (0.35, 0.73)	<0.001

Variables excluded from the model for co-linearity: aminoglycoside resistance, carbapenem resistance, fluoroquinolone resistance, penicillin-β-lactamase inhibitor resistance (co-linear with multidrug resistance); country of origin - United States (co-linear with France, Germany, Italy, and Spain). Variables included but not retained in the model at *P* <0.05: ICU admission, chronic kidney disease, chronic liver disease, country of origin - France.

## Discussion

This international investigation representing the largest cohort study of *Pa*-NP demonstrated high prevalence of MDR at 30.5%. Infection caused by MDR *P. aeruginosa* was found to be an important determinant of hospital mortality, thus, it is critical for clinicians to identify patients at risk of MDR from the onset of infection. Our analysis suggests that the patient’s age, comorbid conditions specifically diabetes, and the severity of infection as indicated by the need for ICU admission predicts infection with a MDR strain of *P. aeruginosa*.

The prevalence of MDR *Pa*-NP is variable depending on the type of study performed and the participating institutions. A recent large epidemiologic study from the United States identified 205,526 *P. aeruginosa* isolates (187,343 pneumonia; 18,183 bloodstream infection (BSI)) and 95,566 *Enterobacteriaceae* specimens (58,810 pneumonia; 36,756 BSI) associated with infection [1]. Prevalence of MDR *P. aeruginosa* (MDR *Pa*) was approximately 15-fold greater than carbapenem-resistant-*Enterobacteriaceae* in both infection types. A net rise in MDR *Pa* as a proportion of all *P. aeruginosa* infections occurred from 2000 to 2009. Likewise, data from the National Healthcare Safety Network (NHSN) in the United States revealed an increased prevalence of MDR *Pa* VAP from the period 2007 to 2008 to the period 2009 to 2010, but, it should be noted the overall prevalence of MDR *Pa* was 17.7% in the latter time period, markedly less than our study [3]. The international composition of the participants is the most likely explanation for the higher prevalence of MDR strains in our study.

The literature also varies with respect to the outcomes of patients with MDR *Pa-*NP. Peña *et al*. examined a Spanish cohort of 91 episodes of ventilator-associated pneumonia (VAP) in 83 patients, 31 caused by susceptible *P. aeruginosa* and 60 by MDR *Pa* strains [18]. These investigators found that susceptible *P. aeruginosa* infections were more likely than MDR *Pa* episodes to receive AIAT and definitive antimicrobial therapy, and in a logistic regression model IIAT was identified as an independent risk factor for early mortality. A recent meta-analysis supports these findings by demonstrating that MDR status is an important determinant of mortality due to nosocomial infections attributed to Gram-negative bacteria, where *P. aeruginosa* and *Acinetobacter* species were the most common isolates [19]. Di Pasquale *et al*. recently found MDR status was not associated with a higher rate of ICU or hospital mortality in patients with ICU-acquired pneumonia. However, unlike our study, the etiology of infection was a mix of Gram-positive and Gram-negative pathogens and there was a small number of MDR *Pa* cases (n = 18) [20].

Increasing antimicrobial resistance in *P. aeruginosa* infections seems to be the most important predictor of outcome. In a recent Brazilian study of *P. aeruginosa* bacteremia isolates from 120 patients [21], 45.8% were resistant to carbapenems, and 23.3% expressed a metallo-β-lactamase gene, *bla*SPM-1 (57%) or *bla*VIM-type (43%). Cefepime-resistance, MDR status and XDR isolates were independently associated with IIAT, which was an important predictor of mortality. These studies support the importance of appropriate and timely antibiotic therapy as a potential determinant of outcome for serious infections attributed to *P. aeruginosa.* Given the association of antibiotic resistance with increasing administration of IIAT and greater hospital mortality, several strategies have been developed to improve upon the appropriateness of empiric therapy in patients at risk of infection with *P. aeruginosa* and other antibiotic-resistant pathogens.

A number of investigations have identified risk factors and scoring systems for infection with MDR pathogens, including MDR *Pa* [22-25]. Major limitations of such approaches are that the potential for IIAT remains, although potentially diminished, and the resultant overuse of broad-spectrum antibiotics in many patients because of the non-specificity of the scoring systems. Novel methods to improve early identification of pathogens and antibiotic susceptibilities are also entering the diagnostic arena. Such diagnostic technology advances offer the potential to maximize administration of appropriate antibiotic therapy while minimizing unnecessary antibiotic exposure. These approaches include the use of molecular methods (for example, polymerase chain reaction electrospray ionization mass spectrometry and matrix-assisted laser desorption/ionization time-of-flight (MALDI-TOF), as well as advanced automated microscopy techniques that allow the identification of bacterial species, the presence of antibiotic resistance genes, and bacterial killing by specific antibiotics within 4 to 6 hours using direct specimen inoculation [26,27].

Our study has a number of limitations. As a retrospective cohort, it is prone to several forms of bias, most notably selection bias. We attempted to mitigate this by enroling consecutive patients fitting the predetermined enrolment criteria. Although we adjusted for known confounders, the possibility exists that some residual confounding remains, particularly confounding by indication. Another important limitation is the potential for patients to be enroled who did not have true pneumonia. Our use of clinical criteria along with microbiologic confirmation was an attempt to maximize the number of patients with *Pa*-NP in our cohort. Additionally, antimicrobial susceptibility testing was performed at the local hospital level. Therefore, the determination of MDR status may have varied more than if a single reference laboratory was used to determine the presence or absence of drug resistance. It is also important to note that although our results strongly suggest that the association of MDR status with increased risk of death is mechanistically related to the risk of receiving inappropriate empiric therapy, we cannot rule out that MDR *Pa*-NP may exert its lethal effect directly due to higher virulence, as has been suggested with other pathogens exhibiting higher minimum inhibitory concentration (MIC) to certain antimicrobials [28,29]. Because we examined hospital mortality rather than the more standard 28-day mortality as the primary outcome for our study, we may have overestimated the magnitude of this outcome. Last, individual antibiotics are commonly part of a regimen for the treatment of nosocomial pneumonia, therefore, independent analysis of the impact on AIAT may not represent the true prescribing practice at each site.

## Conclusions

In summary, our study sheds light on variables associated with to MDR *Pa*-NP; namely decreasing age, diabetes mellitus and ICU admission. In addition, MDR status is an independent predictor of hospital mortality in patients with *Pa*-NP. Given the high rates of MDR *Pa*-NP, advances in rapid diagnosis and susceptibility analysis are needed to direct antibiotic treatment and potentially improve outcomes.

## Key messages

- Among patients with *Pa*-NP, presence of infection with MDR strains is an important independent predictor for hospital mortality.
- Independent predictors of MDR strains of *P. aeruginosa* in this study included decreasing age, diabetes, and ICU admission.
- Advances in rapid diagnostics and antibiotic susceptibility analysis are needed to direct antibiotic treatment and potentially improve outcomes of patients infected with MDR strains of *P. aeruginosa*.

## Abbreviations

AIAT: appropriate initial antibiotic therapy
AOR: adjusted odds ratio
BAL: bronchoalveolar lavage
BSI: bloodstream infection
CDC: Centers for Disease Control and Prevention
CLSI: Clinical Laboratory and Standards Institute
ECDC: European Center for Disease Prevention and Control
ESBLs: extended spectrum β-lactamases
EUCAST: European Committee on Antimicrobial Susceptibility Testing
HR: hazard ratio
IIAT: inappropriate initial antibiotic therapy
MALDI-TOF: matrix-assisted laser desorption/ionization time-of-flight
MDR: multidrug-resistant
NP: nosocomial pneumonia
*Pa*-NP: *Pseudomonas aeruginosa* nosocomial pneumonia
XDR: extensively drug-resistant
